# Developmental suppression of schizophrenia-associated miR-137 alters sensorimotor function in zebrafish

**DOI:** 10.1038/tp.2016.88

**Published:** 2016-05-24

**Authors:** J Giacomotto, A P Carroll, S Rinkwitz, B Mowry, M J Cairns, T S Becker

**Affiliations:** 1 Brain and Mind Research Institute, Sydney Medical School, University of Sydney, Camperdown, NSW, Australia; 2 Psychiatric Genomics Group, Queensland Brain Institute, The University of Queensland, Brisbane, QLD, Australia; 3 School of Biomedical Sciences and Pharmacy, Faculty of Health and Medicine, The University of Newcastle, Callaghan, NSW, Australia; 4 Queensland Centre for Mental Health Research, University of Queensland, Brisbane, QLD, Australia; 5 Schizophrenia Research Institute, Sydney, NSW, Australia

## Abstract

The neurodevelopmentally regulated microRNA miR-137 was strongly implicated as risk locus for schizophrenia in the most recent genome wide association study coordinated by the Psychiatric Genome Consortium (PGC). This molecule is highly conserved in vertebrates enabling the investigation of its function in the developing zebrafish. We utilized this model system to achieve overexpression and suppression of miR-137, both transiently and stably through transgenesis. While miR-137 overexpression was not associated with an observable specific phenotype, downregulation by antisense morpholino and/or transgenic expression of miR-sponge RNA induced significant impairment of both embryonic and larval touch-sensitivity without compromising overall anatomical development. We observed miR-137 expression and activity in sensory neurons including Rohon–Beard neurons and dorsal root ganglia, two neuronal cell types that confer touch-sensitivity in normal zebrafish, suggesting a role of these cell types in the observed phenotype. The lack of obvious anatomical or histological pathology in these cells, however, suggested that subtle axonal network defects or a change in synaptic function and neural connectivity might be responsible for the behavioral phenotype rather than a change in the cellular morphology or neuroanatomy.

## Introduction

MicroRNAs (miRNAs or miRs) play an active role in the regulation of gene expression by modifying mRNA stability and translation, usually through specific albeit partial complementary binding to the 3′ untranslated region.^1, 2^ Most miRNAs are predicted to regulate several hundred mRNAs, and many mRNAs are regulated by multiple miRNAs.^3^ These molecules play a crucial role in development, particularly in the brain, where they are believed to be particularly important for stabilizing gene-regulatory networks during the transition between developmental states.^4, 5, 6^ The idea, however, that miRNAs may play an important role in some human disease/disorders is gaining momentum, and as these molecules have the ability to silence many genes simultaneously, dysregulation of even a single miRNA can have a significant polygenic effect.

Schizophrenia (SCZ) is a psychiatric disorder with a lifetime prevalence of about 1%.^7^ While its etiology is poorly defined there is now substantial support for a complex polygenic inheritance model involving interplay between genetic and environmental factors.^8, 9, 10^ SCZ is characterized by sensory, cognitive and neuroanatomical abnormalities, which may have a developmental origin relating to dysregulation of gene expression. Small non-coding RNAs in particular have emerged as potential candidates as they can exert a broad influence on functional networks and have been found to be dysregulated in SCZ postmortem brains.^11, 12, 13, 14^ The possibility that miRNA may also be directly involved in the pathogenesis of SCZ was highlighted in the most recent international genome wide association studies coordinated by the Schizophrenia Working Group of the PGC.^15^ These studies strongly implicate the *MIR137* locus in the susceptibility for schizophrenia and suggest that the encoded miRNA may be involved in the disorder through a role in shaping neurodevelopment.^1, 16, 17, 18, 19^ MiR-137 is highly expressed in the brain^20, 21^ and has been associated with neuronal differentiation, maturation and transmission, dendritic spine morphogenesis and synaptogenesis.^20, 21, 22, 23, 24^ It has also been linked to murine adult neural stem cell proliferation and differentiation.^25^ While miR-137 seems to have important diverse biological functions, particularly in the brain, this has not yet been fully elaborated in molecular or genetic intervention studies *in vivo.* Model systems capable of revealing cellular, neuroanatomical and neurobehavioural dimensions of miR-137 will be vital to galvanize the emerging evidence for a role in neuropsychiatric and neurocognitive disorders.

The zebrafish (*Danio rerio*) is an attractive and versatile model to gain insight into vertebrate brain development and function. Zebrafish larvae and embryos present a variety of early behaviors such as spontaneous swimming behavior and specific motor responses to diverse stimuli (such as light, chemical or mechanical stimulation) that all result from complex and specific neuronal interactions, thus allowing easy interrogation of brain functions and integrity.^26, 27, 28, 29, 30, 31, 32, 33, 34^ Due to its transparency and extra-uterine development, the zebrafish is also versatile for large-scale experiments and neuroanatomical analysis using conventional microscopy.^28, 34, 35, 36, 37^ This model was pivotal in revealing the critical role of miRNAs in the development of the vertebrate brain.^32^ Several more recent studies have also successfully demonstrated *in vivo* the specific role of candidate miRNAs in nervous system development and function.^34, 38, 39, 40^ Here, we use the zebrafish to investigate the neuroanatomical and neurobehavioral function of miR-137 during development. This was achieved by the modulation of miR-137 expression in the zebrafish embryos throughout early development by both (i) direct administration of synthetic miR-137 mimic or morpholino (MO) miR-137 antagonist, and (ii) inducible transgenic expression of miR-137 or transgenic anti-miR137 sponge-RNA expression. While upregulation produced no observable specific phenotype, both transgenic miR-sponge and MO-induced downregulation of miR-137 inhibited touch–response behavior at embryonic and larval stages without modifying other swimming behaviors. We found that miR-137 in zebrafish is expressed and active in the sensory neurons responsible for touch-sensitivity of zebrafish embryos and larvae. However, no obvious anatomical abnormalities were detected, suggesting subtle defects or a possible change in synaptic function or overall activity.

## Materials and methods

### Zebrafish maintenance and transgenic lines

Adult Zebrafish and embryos were maintained by standard protocols approved by the University of Sydney Animal Ethics Committee.

### MOs and synthetic miRNA injections

MOs used in this study were obtained from Gene Tools (http://www.gene-tools.com) and dissolved following manufacturer's instructions. MOs were injected into one- to four-cell-stage embryos. The appropriate concentration (100 μm to 2000 μm final) was mixed with phenol red in a 4 μl mix, and 1 nl was injected into the yolk just under the cell(s) in order to deliver 1–16 ng depending on the experiments. miR-137 mimics was ordered from Sigma-Aldrich (Sigma Genosys, USA, miR-137 duplex) and miR-Control was mirVana miRNA mimic Negative Control #1 from Applied Biosystems (Cat# 4464059), both were dissolved following manufacturer's instructions. Synthetic miRNAs were injected in a range of 0.025–0.15 ng per embryos as described for MO injections. All MOs and synthetic miRNA sequences used in this study are listed in Supplementary Table 1. miR-430 mimics was obtained from Ambion (product ID MC10393) and was co-injected along with miR-137 mimics at 100 pg.

### Anti-miR137 sponge (SP137) construct and transgenic lines

**Figure 1:**
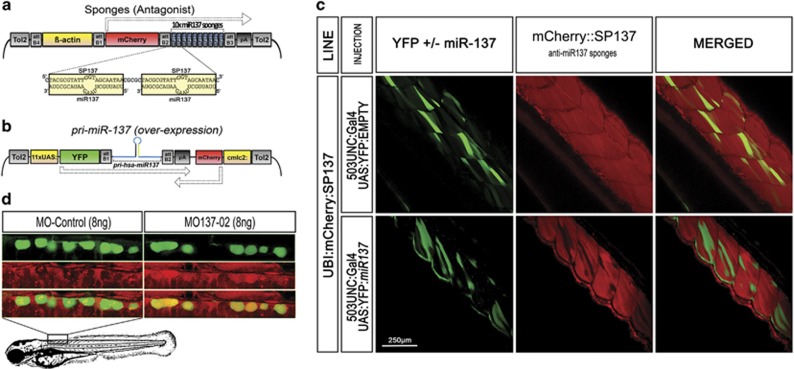
Generation and validation of molecular tools used to transgenically inhibit or overexpress miR-137. (**a**) Transgene used to ubiquitously express anti-miR137 sponges (named βactin:mCherry:10 × SP137). (**b**) Transgene used to overexpress synthetic-miR137 (named UAS:YFPs:miR137). (**c**) Validation of synthetic-miR137 and anti-miR137 sponge transgenic expression/activity in 3 dpf zebrafish. Transgenic animals βactin:mCherry:10 × SP137, which ubiquitously express mCherry:10 × SP137, were injected with two plasmids simultaneously (i) 503UNC:Gal4 expressing Gal4 specifically in muscle cells and (ii) UAS:YFPs:*miR137* expressing YFP fused to synthetic-miR137. Muscle-specific expression of synthetic-miR137 induced by presence of Gal4 can be tracked by the presence of YFP (processed in green in the present pictures), and correlates with downregulation of mCherry fluorescent intensity, confirming efficient activity of both anti-miR137 sponges and synthetic-miR137. Mosaic expression of YFPs:miR137 was observed as the transgenes were injected and thus not stably integrated into the genome. (**d**) Confocal images (0.86-μm section) showing endogenous miR-137 translational repression activity on a transcript carrying anti-miR137 sponges in 3-dpf zebrafish. The injected zebrafish (βactin:mCherry:10 × SP137; SEN:GFP) expressed mCherry:10 × SP137 ubiquitously and GFP specifically in sensory neurons (Rohon–Beard cells presented in **c**. These neurons also expressed endogenous miR-137 (Supplementary Figures 1 and 5). Due to the presence of miR-137, mCherry:10 × SP137 transcript translation was repressed, resulting in poor mCherry expression. Compared with MO-control, injection of MO137-02 (8 ng) dramatically increased fluorescent intensity, confirming that 8 ng MO137-02 was sufficient to inhibit endogenous *miR-137* activity (quantification are presented in Supplementary Figure 5). GFP, green fluorescent protein; YFP, yellow fluorescent protein.

To generate βactin:mCherry:10 × SP137 (Figure 1a), we performed a LR reaction with the following multisite-gateway-compatible clones: 299 (p5E-bactin), 386 (pME-mCherry), homemade p3E-10 × SP137 and destination clone 394 pDestTol2pA2. p3E-10 × SP137 contains 10 × anti-miR137 sponge sequences that were synthetized by Biobasic (https://store.biobasic.com/gene-synthesis/), recognizing both zebrafish and human mature miR-137 (Supplementary Figure 6).

**Figure 2:**
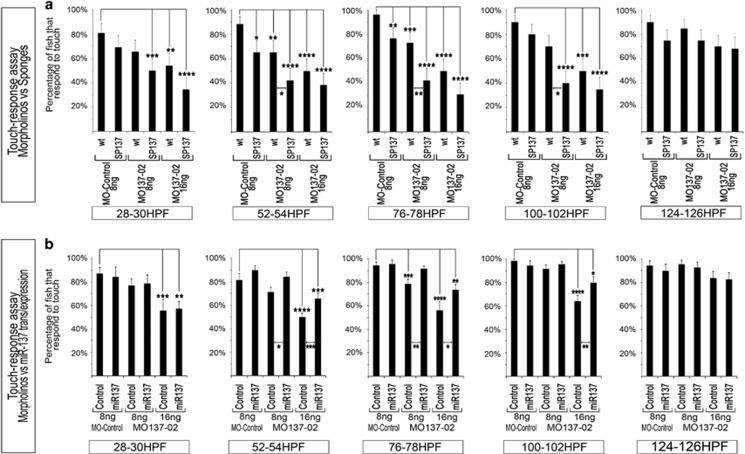
Transgenic anti-miR137 sponge expression acts in synergy with MO137-02, while transgenic pan-neuronal expression of synthetic-miR-137 rescues MO-dependent touch–response behavior. (**a**) Touch–response assay of wild type and transgenic βactin:mCherry:10 × SP137 zebrafish injected with MO-Control or Mo137-02. This assay allows comparing the synergistic effect of anti-miR137 sponges and Morpholinos. (**b**) Rescue experiments based on transgenic pan-neuronal synthetic-miR-137 expression. UAS:YFPs:*miR137* fish were outcrossed with Huc:Gal4; UAS:mCherry animals and embryos were injected with MO137-02 or MO-control. Embryos were sorted at 28 hpf based on presence or absence of YFP expression, and thus on the presence or absence of synthetic miR-137. Touch–response assay was then performed using the selected animals. All experiments were performed in TAB wt background. Significantly different from control at *P<0.05, **P<0.03, ***P<0.02, ****P<0.01 (Student *t*-tests). MO, morpholino; YFP, yellow fluorescent protein.

βactin:mCherry:10 × SP137 was injected into one-cell stage wild-type zebrafish embryos (2 nl of mixture containing 25 ng μl^−1^ of DNA construct along with 20 μg μl^−1^ of transposase RNA to promote DNA integration). Fifty embryos expressing mCherry were sorted and raised to adulthood. Adults were outcrossed with wild types to identify F0 founders that give birth to embryos with strong red fluorescent expression, thus of anti-miR137 sponges. Three independent F1 lines were raised and analyzed to select the line that generated the brightest and most homogeneous transgenic embryos. Those fishes were incrossed to generate the F2 transgenic line that was used in this study to produce the embryos tested in Figure 2c. For this purpose, F2 adults were incrossed and the brightest 20 embryos (similar level of mCherry expression) were selected at 28 hours post fertilization (hpf) to proceed with behavioral analysis.

### Synthetic miR-137 construct and transgenic lines

Synthetic hsa-pri-miR137 was amplified from human DNA using primers 15-Forward-miR137 and 16-Reverse-miR137 (Supplementary Table 1). PCR product was introduced in pDONR221 gateway compatible clone via BP clonase reaction. Synthetic hsa-pri-miR137 was then inserted in 107-UAS-YFPs-Gtwy-clmc2CHERRY via LR clonase reaction. 107-UAS-YFPs-Gtwy-clmc2CHERRY is a modified version of pBH-UAS-YFP-Gtwy from the laboratory of Nonet, in which the yellow fluorescent protein (YFP) stop codon was restored.

107-UAS-YFPs-miR137-clmc2CHERRY was injected in one-cell stage, wild-type embryos using 2 nl of a mixture containing 25 ng μl^−1^ of DNA construct along with 20 μg μl^−1^ of transposase RNA. Embryos were sorted based on mCherry expression in the heart, and 50 embryos were raised to adulthood. F0 founders were identified via outcross with wild types, based again on fluorescent expression in the heart of F1 embryos. The three best F0 founders were selected based on both transmission and fluorescent intensity and were used to generate F1 transgenic lines (via outcross with wild type). F1 transgenic lines were then outcrossed with a Huc:Gal4; UAS:mCherry line that expresses Gal4 in all differentiated neurons. The F1 line that led to the strongest YFP expression (thus of synthetic miR-137) in the nervous system was selected and used in this study. It was named UAS:YFPs:137.

### *In situ* hybridization

miRCURY miR-137 LNA probe for miRNA detection was obtained from Exiqon and prepared following the manufacturer's instruction (http://www.exiqon.com). miR-137 LNA probe was labeled with 5′-DIG and 3′ DIG (reference 35112–15). *In situ* hybridization was performed as described previously.^41^

### Immunohistochemistry

Whole mount immunostaining was performed as previously described.^42^ As primary antibodies, we used anti-GFP (AMS Biotechnology cat#TP401) at 1 : 1000. As secondary antibodies, we used Alexa Fluor 488 (A-11034) from Life Technologies.

### Confocal imaging and fluorescent quantification

Animals were embedded in 1% low melting agarose. For *in vivo* analysis, animals were anesthetized with tricaine. Images presented in this study were acquired using a Zeiss LSM710 confocal microscope coupled with ZEN software. Images were processed under Image J environment when required. Note that YFP signal was processed in green color in the figures presented in this manuscript. When required raw fluorescent intensity was quantified using Image J.

### Behavioral analysis

To obtain staged embryos, male and female zebrafishes were bred at the same time for 1 h. Eggs were collected, washed and distributed as required. When required, embryos were manually dechorionated prior to analysis.

### Touch–response assay

Animals of different ages were observed under a binocular microscope, and touch-evoked response was induced by light mechanical stimuli on trunk or tail. Fish reacting to touch by presenting a swimming response were scored 1 while fish that did respond were scored 0. Twenty embryos per condition were scored, and each scoring was repeated five times.

### Response to flash of light assay

Response to flash of light assay was performed as previously described.^43, 44^ The assay consists of a recording of 30 s with two consecutive flashes of light (~60 000 lumens) at 10  and 20 s. Normal behavior results in a swimming response after the first flash of light, while the second flash is within a refractory period and should not induce any response. Twenty embryos were tested per condition, and repeated three times.

## Results

### Transient manipulation of miR-137 activity

**Figure 3:**
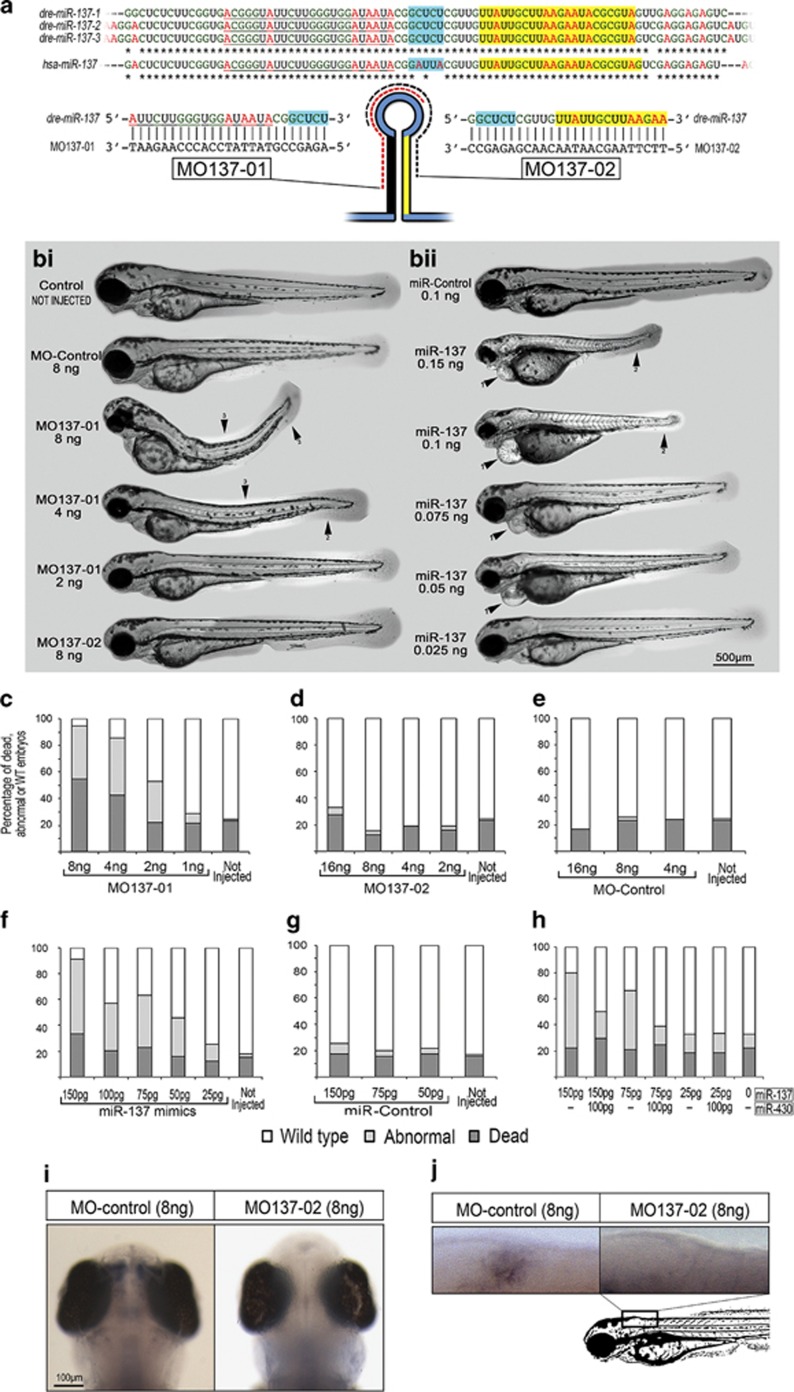
Transient manipulation of miR-137 activity. (**a**) Design of morpholinos (MO) targeting the three dre-pre-miR137 copies, with mature *miR-137* highlighted in yellow. Alignment was performed using CLUSTAL 2.1. (**b**) (i) Morphology of 72 hpf zebrafish embryos injected with MOs. (**b**) (ii) Morphology of 72 hpf zebrafish embryos injected with synthetic miRNAs. (**c**–**h**) Percentage of dead, malformed and normal embryos at 28 hpf following injections of MOs and synthetics miRNAs. MO137-02, MO-Control and miR-control injections were well-tolerated, while MO137-01 and *miR137-*mimics injections induced strong morphological defects that were most likely due to off-specific effect (see Results). These malformations included, but were not limited to, (1) heart edema, (2) curved tail and (3) abnormal trunk curvature. Percentage of normal embryos is presented in white, abnormal in light gray and dead in dark grey. (**i**–**j**) *In situ* hybridization against dre-miR137 at 3 dpf showing that 8 ng of MO137-02 inhibits endogenous *miR-137* expression in zebrafish to a threshold that is no longer detectable even after extended revelation. miR, miRNA.

To transiently manipulate miR-137 activity in zebrafish, we utilized direct delivery of synthetic miR-137 oligonucleotide antagonists and mimics. MiR-137 suppression was achieved with two MOs, designated MO137-01 and MO-137-02, which were designed to interfere with miR-137 maturation by masking Drosha cleavage sites (Figure 3a, Supplementary Table 1).^45^ These anti-miRs were in theory capable of neutralizing the entire *dre-miR-137* family as all three *dre-miR-137* genes in the zebrafish genome share the exact same pre-miR sequence (Figure 3a). It is noteworthy that the mature miR-137 is fully conserved between zebrafish and mammals including humans, with hsa-pre-miR137 presenting only three mismatches in the pre-miR loop sequence (Figure 3a). For transient miR-137 overexpression, we used a synthetic version of mature miR-137, designated miR-137 mimic (Supplementary Table 1).

**Figure 4:**
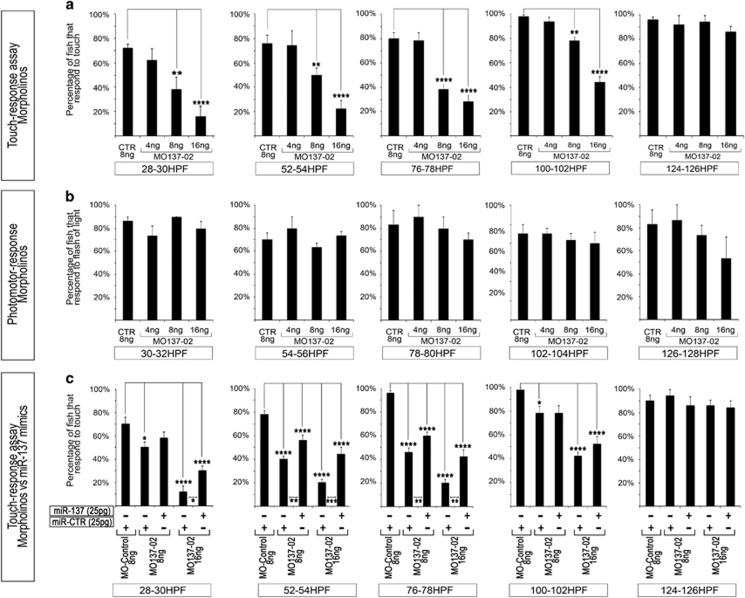
*miR-137* knockdown impacts zebrafish touch–response behavior. (**a**) Touch–response assays following MO137-02 or MO-control injection. (**b**) Response to flash of light assay following MO137-02 or MO-control injection. (**c**) Rescue experiments based on miR137-mimics co-injection with MO. All experiments were performed using the Casper zebrafish strain. Different from control using *t*-test at *0.05, **0.03, ***0.02, ****<0.01. miR, miRNA; MO, morpholino.

We first tested anti-miR137 MOs in zebrafish by injecting a range of doses. While MO137-02 and MO-Control did not induce any obvious morphological defect at the different concentrations tested, MO137-01 injection led to strong anatomical abnormalities (Figure 3b and e). For example, 55% of the embryos died in the first 28  hpf following injection of 8 ng. About 40% presented with strong body deformities (such as heart edema, curved tail and abnormal trunk curvature) and only 5% were considered normal (Figure 3c). These phenotypes persisted at later stage as shown at 72 hpf in Figure 4b. It is noteworthy that these abnormal phenotypes are typical of MO off-specificity, phenotypes also called 'monster phenotypes'.^46^ These typical phenotypes were dose dependent and started to disappear at concentrations <2 ng (Figure 3b and c). It is noteworthy that co-injection of either 75  or 25 pg of synthetic miR-137, as described below, failed to rescue these presumed off-target defects observed in embryos treated with 8 ng of MO137-01. We next tested the effect of different concentrations of synthetic miRNA. We injected miR137-mimics and miR-Control in the dose range of 25–150  pg (Figure 3b, f and g). miR-Control did not induce any obvious morphological defects in the injected embryos. On the contrary, all doses of the miR137-mimics (with the exception of 25 pg) induced strong abnormalities, including growth retardation, small eyes, heart edema and curved tail. High concentrations of miRNA mimics have previously been shown to induce off-target effects with similar morphological defects through non-specific downregulation of miR-430 activity.^47^ To test this hypothesis, we co-injected miR-137-mimics along with miR-430 mimics (Figure 3h). The number of embryos presented with short size, heart edema or curved tail were reduced in the presence of miR-430-mimics, suggesting that these phenotypes were most likely due to suppression of miR-430 activity. As these adverse effects were not observed at 25 pg, we considered this dosage safe for rescuing MO-mediated miR-137 knockdown phenotype in the following experiments.

### Validation of miR-137 inhibition

To validate the efficiency of MO-mediated downregulation of endogenous miR-137, we analyzed control versus MO-treated animals for the presence of mature miR-137 using *in situ* hybridization. In untreated animals, miR-137 is mainly expressed in the nervous system of zebrafish at 3 days post fertilization (dpf), including strong expression in sensory neurons (Supplementary Figure 1). After injection of 8 ng (or higher) of MO137-02, miR-137 expression was no longer detectable even after extended revelation, suggesting potent inhibition (Figure 3i and j). The miR-137 expression signal started to return in animals treated with only 4 ng. No difference to controls was observed at doses <4 ng. It is noteworthy that the alternative miR-137 MOs, MO137-01, at ⩾4 ng led to a reduction of miR-137 *in situ* signal, which was no more obvious at a dosage of ⩽2 ng. However, as presented in Figure 3c, MO137-01 induced strong defects and premature death even down to 4 ng that appeared to be most likely due to off-target effects, thus precluding its application in further experiments as there is no relevant dose that could lead to potent miR137 inhibition without inducing off-specific morphological defects.

### miR-137 modulated sensorimotor phenotype in zebrafish larvae

As presented above, MO137-02 (2 –16 ng) did not induce any obvious morphological defect during early development. However, we found that transient miR-137 knockdown inhibited the touch-evoked escape response of zebrafish larvae (Figure 4). MO137-02 injections at 8 –16 g reduced the response of zebrafish to mechanical stimulus from 28 hpf to 4 dpf, without affecting response to light stimulus or spontaneous movement (Figure 4a and B, Supplementary Videos 1–3). From our experience, we know that cardiovascular or motor neuron malformation can lead to locomotor phenotypes. We analyzed the effect of MO137-02 on those tissues. Both motor neuron- and vascular-development were normal in all conditions tested (Supplementary Figures 2 and 3). To validate these results, we attempted to rescue the MO137-02-induced touch–response phenotype by introducing synthetic miR-137. We co-injected miR137-mimics or miRNA control (miR-CTR) with MO137-02 or MO-control (Figure 4c). MiR-137-mimics partially rescued the touch–response phenotype induced by MO137-02 from 28 to 78 hpf, whereas no significant difference was observed at 4 dpf. In addition to this touch–response phenotype, we also found that animals treated with 16 ng MO presented abnormal death ratios between 6 and 7 dpf. This premature death was not rescued by co-injection of miR-137 mimics, which is not surprising given that these molecules are only likely to remain effective for a few days following injection (Supplementary Figure 4).

### Transgenic manipulation of miR-137 activity

To further validate the phenotypic changes observed by direct chemical manipulation of miR-137 *in vivo*, we also introduced transgene constructs into the zebrafish genome designed either to block miR-137 activity by competing with its endogenous targets (Figure 1a) or to overexpress this small molecule (Figure 1b).^34^ The miR-137 inhibitor construct (Figure 1a) constitutively expressed miR137-target sequence repeats (miR-sponges or SP137) in a fusion mRNA encoding the fluorescent marker protein mCherry.^34, 48^ The sponges were designed to present a bulge at position 9–12 of miR-137 to maximize antagonistic effect without inducing RISC-associated RNA-cleavage of the mCherry:SP137 mRNA (Figure 1a).^37^ The synthetic-miR137-inducible plasmid was constructed by cloning the human pri-miR downstream of the fluorescent marker YFP and under the control of the UAS promoter, thus allowing inducible expression of both YFP and miR-137 in tissue(s) expressing the Gal4 protein (Figure 3b).^49^ Human pri-miR137 was cloned instead of zebrafish pri-miR because of the three mismatches in the loop sequence, making it less sensitive to MO-inhibition and thus improving potential rescue experiments. To be able to select animals that inherited the plasmid without the presence of Gal4 (silent carriers), we used a cmlc2:mCherry cassette that induces expression of mCherry in the zebrafish heart. Transgenic lines were generated and named, respectively, βactin:mCherry:10 × SP137 for the sponge construct and UAS:YFPs:miR137 for the inducible plasmid. To confirm expression and activity of both agonist and antagonist systems, βactin:mCherry:10 × SP137 embryos expressing homogeneously and ubiquitously mCherry and SP137 were injected with both 503UNC:Gal4 (expressing Gal4 in muscle cells)^50^ and UAS:YFPs:miR137 (Figure 1c). Mosaic expression of synthetic-miR137 induced by presence of Gal4 can be tracked by the co-expression of the YFP protein (fluorescence processed in green in Figure 1c). We induced expression in muscles as this tissue does not express endogenous miR-137, which would have interfered with our readout. Synthetic-miR137 expression correlated with downregulation of mCherry fluorescent intensity, confirming efficient activity of both the sponges and the transgenic synthetic-miR137 (Figure 1c).

We also took advantage of the βactin:mCherry:10 × SP137 transgenic line, which expresses mCherry:10 × SP137 RNA ubiquitously, to (i) validate SP137 affinity for endogenous miR-137, (ii) confirm presence and activity of endogenous miR-137 in specific cell types and (iii) validate MO efficiency in these tissues. In addition to having the potential to block miR-137 activity by competing with its endogenous targets, mCherry:10 × SP137 RNA can be used as a sensor of miR-137 activity. By binding to SP137, endogenous miR-137 should repress mCherry translation, thus reducing red fluorescence where this miRNA is expressed. We crossed βactin:mCherry:10 × SP137 animals with a SEN:GFP zebrafish line that expresses GFP in, but not limited to, sensory neurons including Rohon–Beard (RB), dorsal root ganglia (DRG) and trigeminal neurons (TR), as well as non-sensory neurons Mauthner cells (M); cell types that were found to express endogenous miR-137 by *in situ* hybridization. As seen in Figure 1d, due to the presence of miR-137, GFP-positive RB cells presented weak red fluorescence, which was increased dramatically in the presence of 8 ng of MO137-02. Similar results were obtained with M and DRG cells, but not with motor neurons or muscle cells (Supplementary Figure 5). These observations demonstrate that endogenous miR-137 is specifically expressed and functional in RB, M and DRG cells, and that MO137-02 efficiently reduces its activity.

### Anti-miR-137 sponge reduces zebrafish touch–response

Zebrafish βactin:mCherry:10 × SP137 transgenic animals expressing SP137 presented not only a similar attenuated touch–response phenotype as observed following MO137-02 injection, but they also potentiated the effect of this MO (Figure 2a). Indeed, embryos expressing anti-miR137 sponges were significantly more sensitive to MO-mediated miR137 knockdown from 2 to 4 dpf. This synergistic effect observed between Sp137 and MO137-02 supports the association of miR-137 suppression with decreased touch-sensitivity in zebrafish embryos and larvae. We then tried to rescue this phenotype through neuron-specific transgene expression of the miRNA using the pan-neuronal Huc promoter.^51^ This was achieved by crossing the UAS:YFPs:137 transgenic line, that carries the construct presented in Figure 1b, with a Huc:Gal4 driver line available in our laboratory. Embryos were injected with either MO137-02 or MO-control, and sorted at 28 hpf for the presence or absence of YFP in the nervous system; a marker for the presence or absence of transgenic miR-137 expression. Significantly, this experiment supported the role for miR-137 in touch-sensitivity observed in the transient assays, as transgenic expression of miR-137 in neurons was sufficient to partially rescue the touch-insensitivity of MO137-02 injected embryos (Figure 2b). It is noteworthy that transgenic pan-neuronal expression of miR-137 increases the response of the fish in all conditions tested except at 28 hpf. This exception may be due to the fact that the Huc promoter (which drives expression of the transgene) is only active in differentiated neurons. In contrast, the miR137-mimic was present from one-cell stage but may lose its effect at 4 dpf because of breakdown and dilution during development of the growing animal. Transgenic miR-137 is still expressed at 4 dpf though. Finally, we did not record obvious abnormal death ratios in βactin:mCherry:10 × SP137 embryos, while transgenic pan-neuronal expression of human miR137 reduced the number of death events in MO137-02 (16 ng)-injected embryos between 6 and 7 dpf (Supplementary Figure 4).

### miR-137 knockdown does not induce obvious anatomical defects of the sensory neurons

**Figure 5:**
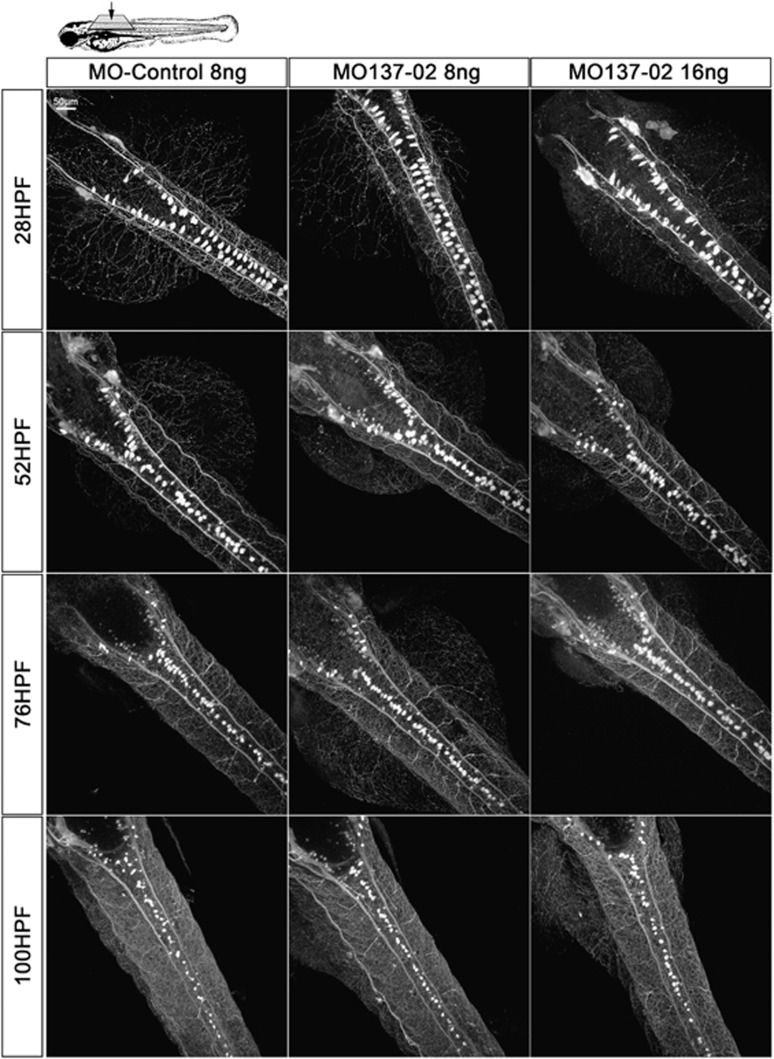
miR-137 knockdown does not impair Rohon–Beard- (RB-), dorsal root ganglia- (DRG-), trigeminal- or Mauthner-(M-)neurons differentiation and seems to not modify their overall network. SEN:GFP transgenic animals were injected with 8–16 ng of MO-Control or MO137-02 to observe RB, M and DRG cells *in vivo*. No significant difference with regard to the number of cells was observed between the different conditions (Counting available in Supplementary Figure 7). No obvious difference was observed in terms of axonal network, but an in-depth analysis should be performed to conclude. Following anti-GFP immunostaining, 10 animals per conditions were analyzed at different time points using confocal microscopy and image analysis. Brightness of the original image was enhanced. GFP, green fluorescent protein; MO, morpholino.

Considering that inhibition of miR-137 inhibits the response of zebrafish embryos and larvae to mechanical stimuli, and that this miRNA is expressed in sensory neurons, we hypothesized that miR-137 might be involved in the early development of these cell types. We took advantage of the SEN:GFP line that expresses GFP in sensory neurons. We injected MO-control and MO137-02 into one-cell stage SEN:GFP embryos and observed development of RB, DRG, TR and M neurons (Figure 5, Supplementary Figure 7). Surprisingly, no obvious difference in the number of cells or their development was detected between the different conditions. Similarly, no obvious difference in term of axonal network was detected in the different animals analyzed. These observations suggest that miR-137 knockdown inhibits the touch response of zebrafish embryos and larvae without affecting RB, DRG, TR or M cell differentiation or their axonal projections. It is noteworthy that to fully conclude on the absence of axonal network defect, a more in-depth analysis would be required as we cannot exclude subtle changes.

## Discussion

Large-scale genetic association studies of schizophrenia have recently provided exciting new loci that have the potential to transform our thinking about the etiology of the disorder. Among these discoveries, the gene for the small non-coding RNA miR-137 was implicated with the second highest association (*P*<10^−17^).^52^

While this extraordinary regulatory molecule has the potential to influence many developmental and neurological processes, to better understand their implications *in vivo*, we took advantage of the zebrafish model to investigate its function in the developing vertebrate nervous system. For this purpose, we designed several MOs and tested both their capacity to suppress miR-137 maturation and their toxicity. One of these molecules, MO137-02, was able to inhibit miR-137 with no obvious side-effects during early development. This miRNA antagonist was also able to induce a robust behavioral phenotype characterized by a profound reduction in touch-sensitivity. Importantly we were able to partially rescue this phenotype by co-administration of synthetic miR-137 and by transgenic pan-neuronal expression of miR-137. Interestingly, at high dose (16 ng) of this MO, we also saw significant lethality that we initially attributed to non-specific toxicity; however, in view of the recent observation of embryonic lethality in the miR-137 homozygous knockout mouse^53^ it is possible that this dose was ablating miR-137 function and inducing death.

To further support these observations without the associated off-target effects of oligonucleotide-based manipulation, we established a transgenic miR-137 suppression system that worked through the production of a stable miR-137 sponge transcript. This approach accorded well with the neurobehavioral observations generated by the MO as it was able to recapitulate the reduction in touch-sensitivity response and acted synergistically with the MO antagonist. Interestingly, we were not able to reproduce the intensity of the effect in the transgenic animals with that seen in animals treated with the transient antagonist. This can be explained by the fact that the MO is available at high concentrations in the embryos, while the expressed antagonist is available at more modest concentrations. Although there is certainly room for enhancement by increasing the level of sponge expression through the use of a stronger promoter and/or by allowing accumulation of greater transgene copy numbers. Regardless, this is a valuable tool for miRNA inhibition that is not limited to early stages of development. Finally, considering the recent advance of genome editing tools, an interesting option would also be to knockout miR-137.^54^ However, this strategy would require considerable effort, as three different copies of the miR-137 gene exist in zebrafish.

miRNAs are often described as actors of cell proliferation and differentiation, and of transition between these two states. Our first hypothesis was that miR-137 would impact such cellular mechanisms in sensory neurons thus delaying touch–response behavior. While we were able to show that this molecule was expressed quite broadly in the nervous system of zebrafish, including sensory neurons, we did not detect any obvious anatomical abnormality in RB or DRG neurons and their corresponding networks. However, it is possible that miR-137 inhibition would lead to subtle histological changes not detected in this study. It is also possible that its inhibition would modify the synaptic functionality of these cells through the regulation of signal transduction pathways, neurotransmitters and/or ion channels, which have been implicated in bioinformatics and functional screening of miR-137 target genes.^55^ For instance, the l-type voltage-dependent calcium channel CACNAC1, which was recently associated with SCZ,^15, 56^ is present in zebrafish with a transcript predicted to have two putative sites for miR-137. More recently, miR-137 has also been shown to modify presynaptic function though its regulation of *Syt1* in the mouse.^57^ Interestingly, zebrafish present two copies of the *syt1* gene, with both having two putative miR-137 sites in their 3′ untranslated region. In addition, as this small RNA is associated with a number of neuronal biological mechanisms such as proliferation, maturation and transmission, dendritic morphogenesis and synaptogenesis^21, 22, 23, 24, 25^ and that we find that it is expressed in other parts of the brain, we have to consider that the observed touch–response phenotype might be due to different subsets of neurons.

In conclusion, we find that miR-137 knockdown reduces zebrafish touch–response behavior. While this phenotype might not be directly relevant to SCZ, understanding how this miRNA impacts the sensorimotor function in zebrafish larvae may help identify important molecular functions involved in the disease. Indeed, miR-137 is conserved between zebrafish and human, thus its molecular role should have many parallels even if its physiological role may have diverged to some extent. Several studies in SCZ patients have observed abnormal sensory deficits, including pain^58, 59, 60^ and olfactory^61, 62^ deficits. SCZ patients also show consistent deficits in sensorimotor gating in measurements of prepulse inhibition.^63^ This suggests that the sensory miR137-dependent phenotype observed in zebrafish is potentially of high interest to bring new insight into schizophrenia biology. It is probable that miR-137 function, which modulates zebrafish touch–response phenotype, would act through the same mechanisms in other neuronal population(s) to lead to pain- and olfactory-deficits.
