# Supplementary material for: Developmental suppression of schizophrenia-associated miR-137 alters sensorimotor function in zebrafish
Source: Transl Psychiatry. 2016 May 24;6(5):e818–. doi: 10.1038/tp.2016.88 (PMC5070046; doi:10.1038/tp.2016.88)
Supplement: Supplementary Information [file tp201688x4.docx]

**Supplemental materials**


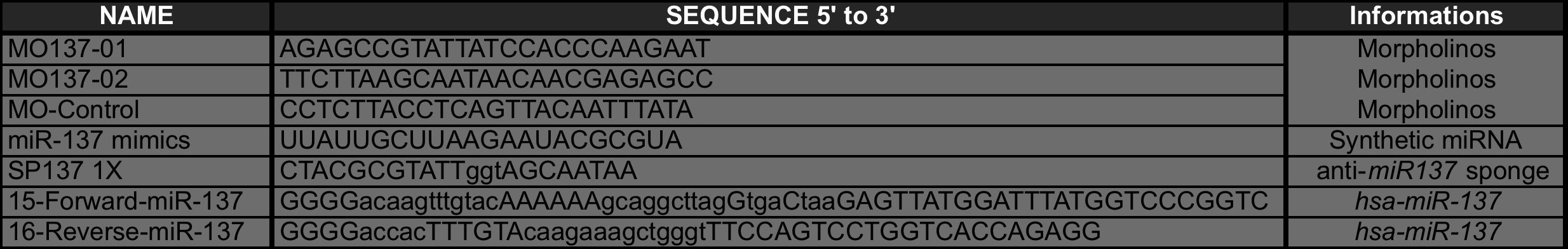


**Supplementary table 01: Primers and oligonucleotide sequences used in this study.**

**
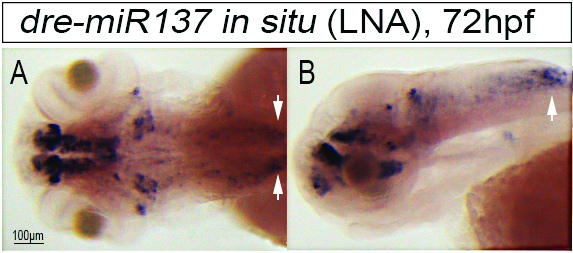
**

**Supplementary figure 01: *dre-miR-137* in situ hybridisation. A-C,** in situ hybridisation using an LNA probe on 72hpf zebrafish embryos. *miR-137* is expressed in the nervous system including in fore-, mid- and hindbrain. miR-137 is also highly expressed in sensory neurons (white arrows).

**
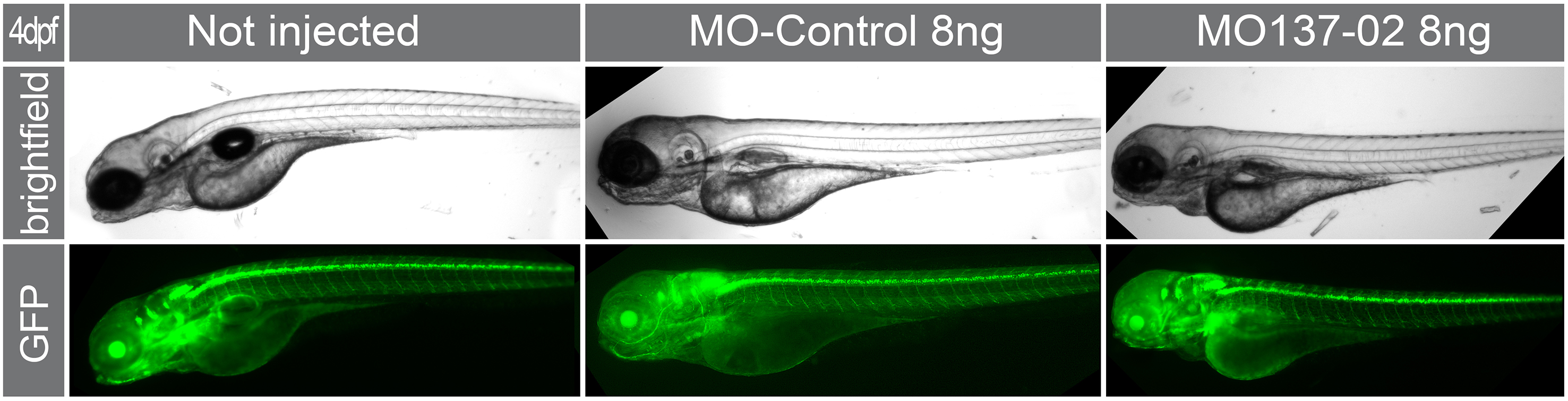
**

**Supplementary figure 02: *miR-137* knockdown does not impact overall motor neuron development.** A transgenic zebrafish line available in our laboratory (MN:GFP) that expresses GFP fluorescent protein into motor neurons was injected with 8ng of MO-Control or MO137-02. No significant difference was observed between the different conditions.

**
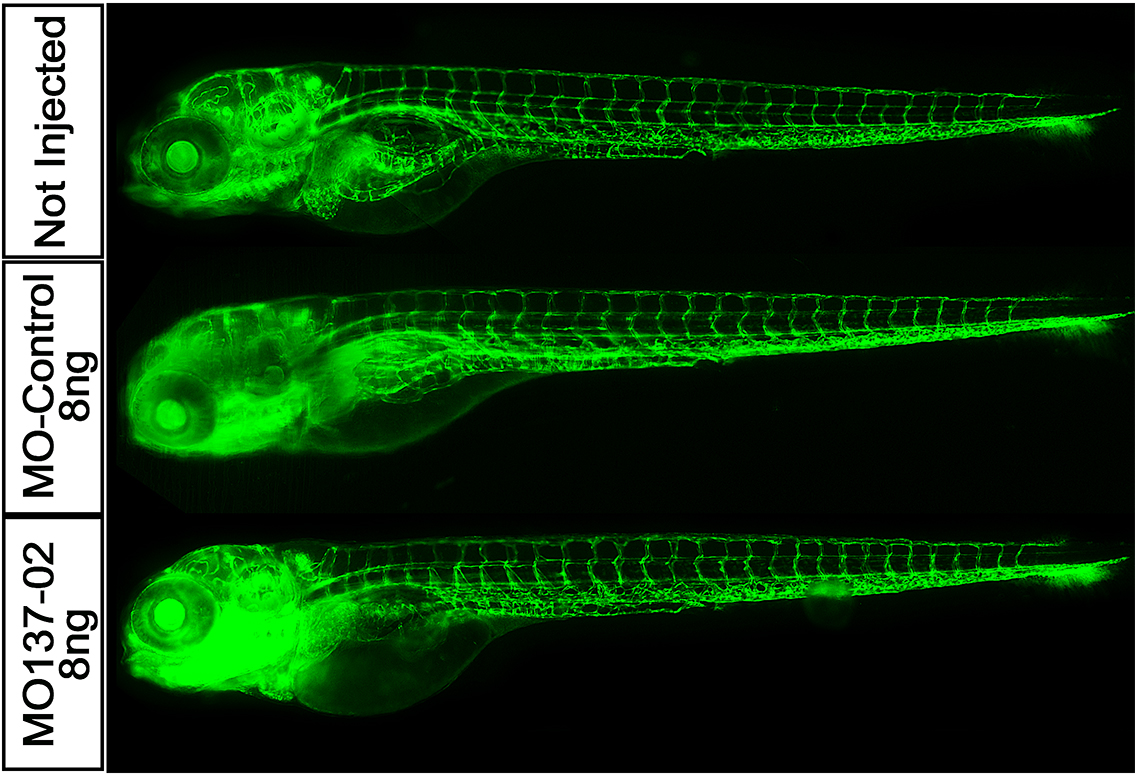
**

**Supplementary figure 03: *miR-137* knockdown does not impact overall vascular system development.** Fli1:GFP zebrafish (4dpf) expressing GFP into the vascular system was injected or not with 8ng of MO-Control or MO137-02. No significant difference was observed between the different conditions.


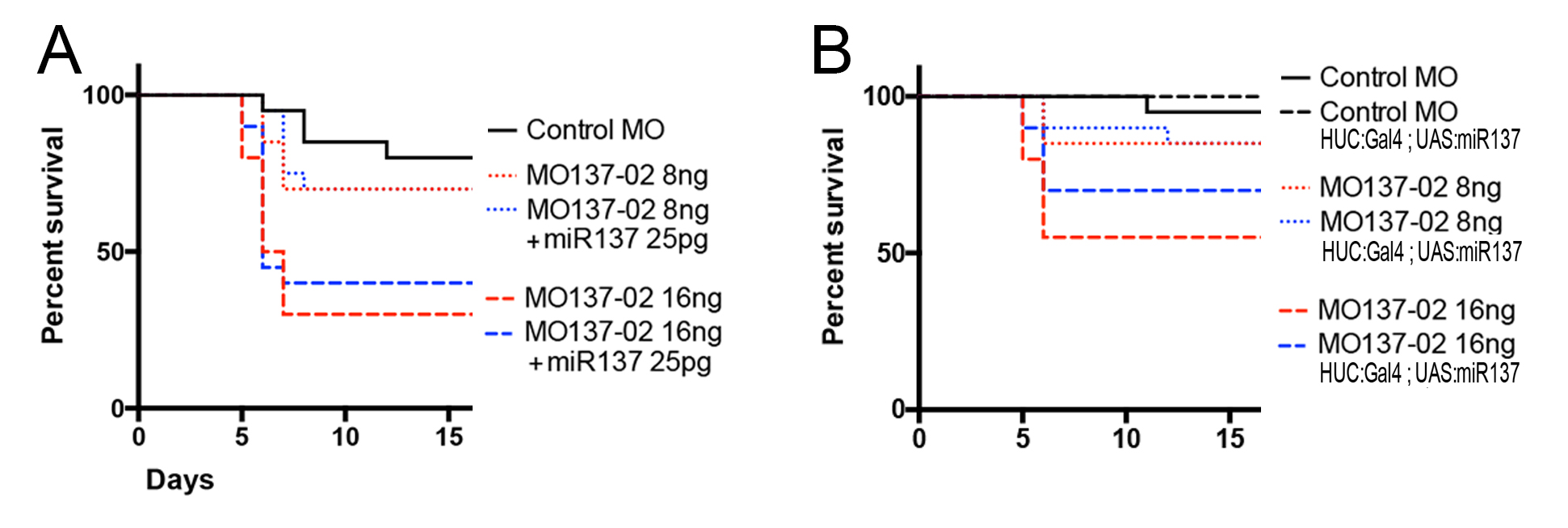


**Supplementary figure 04: Survival assay.** A, Experiment performed in Casper strain background. B, Experiment performed in TAB wt background in presence or absence of Huc:Gal4 ; UAS:YFP:miR137 transgene (inducing pan-neuronal expression of miR137).

**
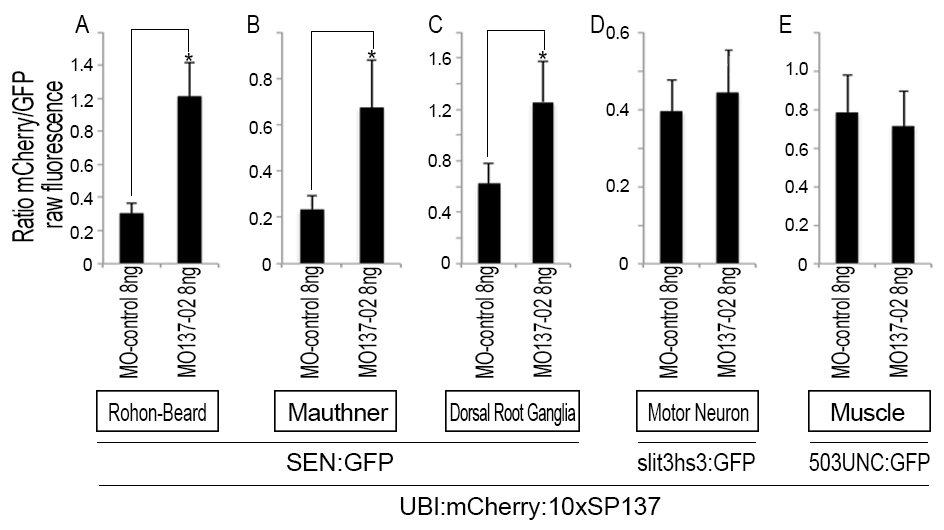
**

**Supplementary figure 05: Evaluation of endogenous miR-137 activity using βactin:mCherry:Sp137 transgene as reporter, and validation of MO137-02 knockdown efficiency.** βactin:mCherry:10xSP137 transgenic line expresses mCherry:10xSP137 RNA ubiquitously. If present, miR-137 should bind to anti-miR-137 sponges (SP137) and should repress mCherry translation, thereby reducing red fluorescent expression in tissues where this miRNA is expressed. Different transgenic lines were used as markers: SEN:GFP transgenic line expressing GFP in different tissues including Rohon-Beard and trigeminal neurons, Mauthner cells and Dorsal Root Ganglia. MN:GFP and 503UNC:GFP expressing GFP in motor neurons and muscle cells respectively. We outcrossed βactin:mCherry:10xSP137 with those line, allowing us to track these tissues and evaluate miR-137 translational repressing activity on mCherry:10xSP137 RNA. Injection of MO137-02 at 8ng did not significantly modify red fluorescence intensity in motor neurons or muscle cells, suggesting absence of miR-137 activity in these tissues. However MO137-02 significantly increased red fluorescence in RB, M and DRG cells, confirming both presence of miR-137 and efficacy of MO137-02. For technical reasons, we were not able to evaluate fluorescent expression in the trigeminal neurons. Fluorescent quantifications were performed using confocal Zeiss LSM710, with same pinhole and laser intensity for all condition tested. Mean of mCherry/GFP raw fluorescence ratio of 20 measurements (4 measurements on 5 different 3dpf larvae per condition). Different from control at *<0.01.


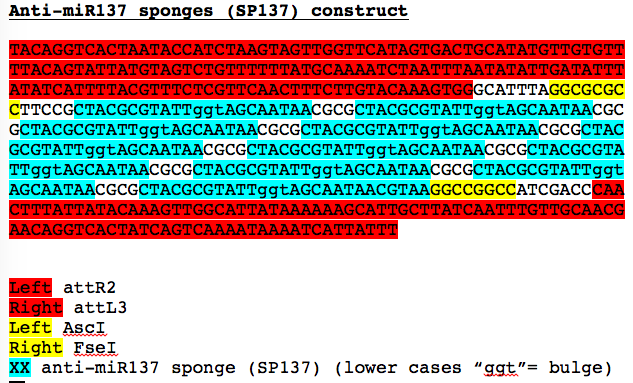


**Supplementary figure 06: Details of SP137 sequence used to generate** **p3E-10xSP137.**

**
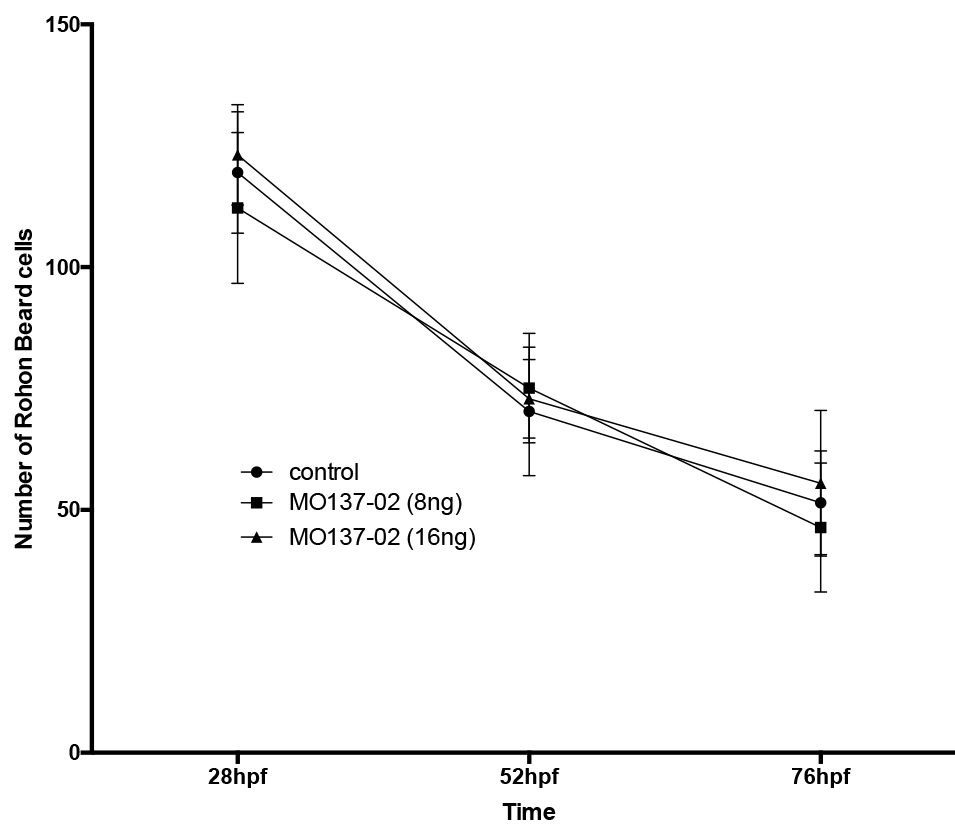
**

**Supplementary figure 07: Number of Rohon Beard cells during zebrafish development after injection of MO-control (8ng) or MO137-02 (8ng and 16ng).** No significant difference in number of cell per time point was observed. Similarly, no difference were observed for DRG, TR and M cells.

**Supplementary video 01: Touch-response assay performed on 3dpf zebrafish larvae injected with 8ng of MO-Control.** 3dpf animals treated with 8ng of MO-control present normal spontaneous activity and response to mechanical stimuli.

**Supplementary video 02: Touch-response assay performed on 28hpf zebrafish larvae injected with 8ng of MO137-02.** 28hpf animals treated with 8ng of MO137-02 present normal spontaneous activity but present reduced or absence of response to mechanical stimuli.

**Supplementary video 03: Response to flash of light assay performed on 3dpf zebrafish larvae injected with either 8ng of MO-Control or 8ng of MO137-02.** 3dpf animals treated with either 8ng of MO-control or 8ng of 8ng of MO137-02 present normal response to bright flash of light. Animal on the left was injected with MO-Control, animal on the right with MO137-02.
